# Utility of endoscopic retrograde cholangiopancreatography in management of pediatric pancreaticobiliary disease

**DOI:** 10.1186/s12887-022-03207-3

**Published:** 2022-03-14

**Authors:** Satoshi Makita, Hizuru Amano, Hiroki Kawashima, Akinari Hinoki, Chiyoe Shirota, Takahisa Tainaka, Wataru Sumida, Kazuki Yokota, Masamune Okamoto, Aitaro Takimoto, Akihiro Yasui, Yoichi Nakagawa, Hiroo Uchida

**Affiliations:** 1 Department of Pediatric Surgery, Nagoya University Graduate School of Medicine, 65 Tsurumai-cho, Showa-ku, 466-8560 Nagoya, Japan; 2 Department of Gastroenterology, Nagoya University Graduate School of Medicine, Nagoya, Japan; 3 Department of Rare / Intractable Cancer Analysis Research, Nagoya University, Nagoya, Japan

**Keywords:** Endoscopic retrograde cholangiopancreatography, Pancreatitis, Congenital biliary dilatation, Stent

## Abstract

**Background:**

The purpose of this study was to evaluate the utility of endoscopic retrograde cholangiopancreatography (ERCP) in pediatric patients with pancreaticobiliary diseases.

**Methods:**

A retrospective review was performed on patients who underwent ERCP for the treatment of biliary tract disease and detailed examination of pancreatitis at our institution from January 1999 to December 2020.

**Results:**

ERCP was performed for congenital biliary dilatation (CBD) (*n* = 42), choledocholithiasis (*n* = 9), common bile duct stenosis (*n* = 1), and several types of pancreatitis (*n* = 13). The only severe complication of ERCP was common bile duct injury. Three (5.8%) of 52 biliary diseases failed to be treated by ERCP. All patients with pancreatic disease were correctly diagnosed and treated.

**Conclusions:**

Endoscopic biliary drainage with a temporary stent was adequate for symptomatic relief in CBD. Stenting of the pancreatic duct was useful for improving the angulation and drainage of the pancreatic duct. ERCP was useful for understanding the anatomy of the pancreatic duct and revealing potential treatments. Therefore, ERCP and transendoscopic therapy are sufficiently feasible in pediatric patients and should be actively introduced for the investigation and treatment of pancreaticobiliary diseases.

## Introduction

The use of diagnostic and therapeutic endoscopic retrograde cholangiopancreatography (ERCP) is steadily increasing in the management of pancreaticobiliary diseases in children [1]. While magnetic resonance cholangiopancreatography (MRCP) is one of the diagnostic imaging modalities available for detecting anatomical abnormalities, MRCP images cannot fully identify anatomical abnormalities in pediatric patients. Therefore, there is a need to identify the scope and utility of ERCP in children with pancreaticobiliary diseases.

We previously performed ERCP in asymptomatic patients with congenital biliary dilatation (CBD) to ascertain anatomical details [2, 3]. Of late, however, ERCP is performed only in symptomatic CBD patients for therapeutic purposes, so as to avoid associated complications.

In addition to CBD, ERCP is also performed for the treatment of choledocholithiasis and for the investigation and treatment of unexplained pancreatitis.

The etiologies of pediatric pancreatitis include drugs, infections, trauma, and anatomical abnormalities. In Japan, Suzuki et al. reviewed 145 pediatric patients with acute pancreatitis and reported that 54.5% of cases were caused by anatomical abnormalities [4]. For patients with unexplained pancreatitis, ERCP and endoscopic transpapillary therapy have been performed as required to detect anatomical abnormalities. The purpose of this study was to evaluate the utility of ERCP in pediatric patients with pancreaticobiliary diseases.

## Patients and methods

 This study was approved by the ethics committee of our institution (#2020 − 0635).

**Fig. 1:**
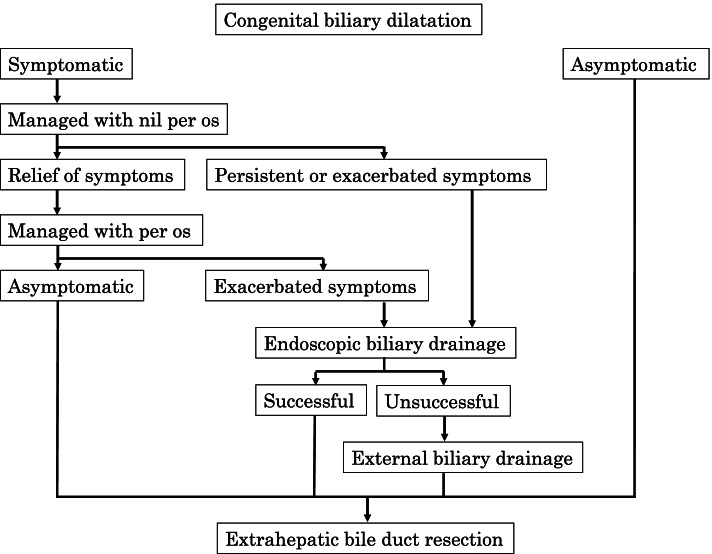
Treatment strategy for preoperative management of congenital biliary dilatation in children

**Fig. 2:**
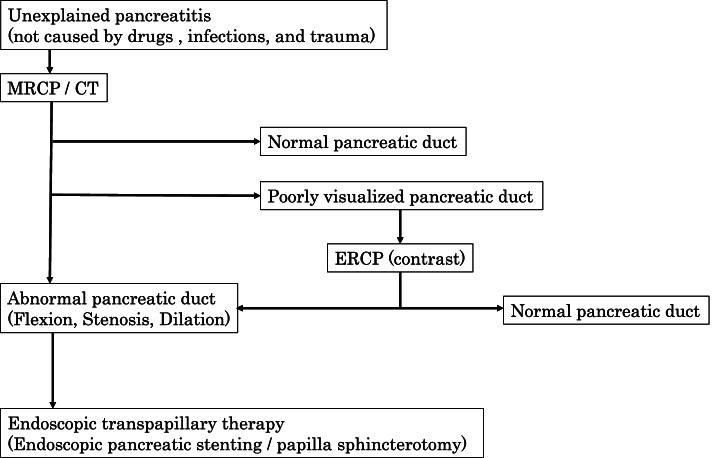
Strategy for investigation of recurrent pancreatitis in children

 Because this was a retrospective observational study and the data analyzed were anonymized, informed consent from participants or their parents/guardians was obtained through an opt-out method on our hospital website in accordance with the Ethical Guidelines for Medical and Health Research Involving Human Subjects in Japan. A retrospective review was performed concerning patients who underwent ERCP for the treatment of biliary tract disease and detailed investigation of the cause of pancreatitis at our institution, from January 1999 to December 2020. Our current treatment strategy for preoperative management of CBD is shown in Fig. 1. Extrahepatic bile resection was performed within 1 month after stenting. The strategy for pancreatitis suspected to be caused by anatomical abnormalities is shown in Fig. 2. ERCP for symptomatic CBD was performed as early as the regular examination schedule permitted after referral to our institution, while ERCP for pancreatitis was scheduled once the clinical symptoms of pancreatitis improved; in both instances, the procedures was performed under general anesthesia. Pancreatitis was defined as the presence of abdominal pain with hyperamylasemia and inflammation. All endoscopic procedures were performed by trained gastroenterologists. For endoscopy, PJF-7.5 (tip outer diameter, 7.8 mm; channel diameter, 2.0 mm; Olympus) and JF-240 (tip outer diameter, 12.6 mm; channel diameter, 3.2 mm; Olympus) were used for infants and school-age children, respectively. The endoscope was selected depending on the body size of the patient and the procedure to be performed. PJF-7.5 was used only for contrast and stent tube insertion. We selected JF-240 for papillotomy since it allowed us to deliver various devices. Endoscopic pancreatic stenting (EPS) was performed for flexion, stenosis, and dilatation of the pancreatic duct to prevent pancreatitis. Stent replacement was performed every three to six months after EPS. Endoscopic transpapillary therapy (stent tube insertion or papillotomy) was attempted when drainage of the bile duct or pancreatic duct drainage was deemed necessary.

## Results

Over the 22-year observation period, ERCP was performed in 52 patients for the treatment of biliary diseases, and in 13 patients for the examination and treatment of pancreatic diseases. These patients comprised 24 boys and 41 girls with an age range of 3 months to 14.8 years (median 4.8 years). We have successfully performed ERCP on a child as young as 3 months and 5 kg.

### Complications after ERCP

Complications due to ERCP were noted in 1 of 65 patients (1.5%). Other than one 2-year-old patient with choledocholithiasis who temporarily required endoscopic nasobiliary tubing for common bile duct injury secondary to ERCP, no other complications, such as severe pancreatitis, bleeding, or perforation, were noted.

### ERCP treatment outcomes by disease

#### Biliary duct disease

##### CBD

The details of ERCP for CBD (*n* = 42) are shown in Table 1. At our institution 194 patients with CBD underwent radical surgery between January 1999 to December 2020. Forty patients were asymptomatic and 154 patients were symptomatic. Six symptomatic patients showed perforation of extrahepatic bile duct, and underwent emergent surgery. The other 110 symptomatic patients relieved their symptoms by nil per os. Of 38 symptomatic patients, placement of a biliary stent tube before radical surgery was successful in 32. All 32 patients who underwent stent insertion were relieved of their symptoms. Removing the protein plug by ERCP in two patients relieved the pain and these patients did not require stenting. Stenting in the other four patients was unsuccessful due to flexion or obstruction of the narrow segment of the biliary tract. Three of the four patients were relieved of their symptoms by means other than stenting. Percutaneous transhepatic biliary drainage in 2 patients (a 5-month-old boy and a 6-month-old boy) and repeated insertion of the guidewire that resulted in crushed protein plug in one patient (a 1-year-old girl) relieved their symptoms. The other patient (also a 1-year-old girl) continued to experience abdominal pain for 6 days until semi-urgent radical surgery.

**Table 1:** Details regarding ERCP for biliary duct disease

Details regarding ERCP	n
**Congenital biliary dilatation **(median age: 3.9 year-old [ 0.4–14.7 ])	
**Preoperative treatment**	
Endoscopic biliary stent	32
Removal of protein plug in the common channel	2
Failure to insert stent (converted to percutaneous transhepatic biliary drainage)	4 (2)
**Intraoperative treatment**	
EPS for intraoperative pancreatic duct injury	1
Pancreatography after pancreatic duct injury repair	1
**Postoperative treatment**	
EPS for intraoperative pancreatic duct injury	1
Removal of protein plug from the pancreatic duct	1
**Choledocholithiasis **(median age: 6.6 years [ 0.3–10.2 ])	
Endoscopic removal of stones by papillary balloon dilatation	9
**Common bile duct stenosis** (age: 11.3 years)	
Endoscopic balloon dilatation of common bile duct	1

*ERCP* endoscopic retrograde cholangiopancreatography

*EPS* endoscopic pancreatic stenting

##### Choledocholithiasis and common bile duct stenosis

Endoscopic removal of common bile duct stones was successfully performed in nine patients with choledocholithiasis. Balloon dilatation was performed for common bile duct stenosis and restenosis was not observed after the procedure.

Three (5.8%) of the 52 biliary disease cases failed to be treated by ERCP owing to difficulties encountered in cannulation.

### Pancreatic disease

A total of 38 ERCP procedures were performed in 13 patients with pancreatic disease. These patients comprised 5 boys and 8 girls with an age range of 1.8 years to 14.8 years (median 7.5 years). The details of ERCP for each patient are presented in Table 2.

**Table 2:** The details regarding ERCP for pancreatitis

Case	Disease	Sex	Age at onset (years)	Age at initial ERCP (years)	No. of ERPs	Details regarding ERCP
1	Pancreatitis after duodenal stenosis surgery with annular pancreas	F	2.8	3.1	3	Contrast imaging − 1 Papillary balloon dilatation + remove stone + EPS 1 Papillary balloon dilatation + remove stone 1
2	Pancreatitis after duodenal atresia surgery with annular pancreas	F	6.5	7.5	6	Contrast imaging 2 Papillary balloon dilatation + EPS 1 EPS 3
3	Pancreatitis after duodenal atresia surgery with annular pancreas	F	1.8	1.8	5	Contrast imaging 1 EPS 4
4	Pancreatitis after duodenal atresia surgery with congenital biliary dilatation	M	1.7	1.8	1	Contrast imaging 1
5	Chronic pancreatitis	F	2.4	3.9	6	Contrast imaging 2 Removal of stone 1 EPS 3
6	Chronic pancreatitis	M	8	12	6	Contrast imaging 1 Removal of stone 1 EPS 4
7	Chronic pancreatitis	M	8	8.3	3	Contrast imaging 2 EPS 1
8	Pancreas divisum	F	4.5	5.2	1	Contrast imaging 1
9	Pancreas divisum	F	5.1	5.8	2	EPS 1 Minor papillotomy 1
10	Autoimmune pancreatitis	F	12.4	12.6	2	Contrast imaging 1 Bile duct stenting 1
11	Hereditary pancreatitis	F	5	8.7	1	Contrast imaging 1
12	Recurrent pancreatitis	M	13	13.8	1	Contrast imaging 1
13	Recurrent pancreatitis	M	14.4	14.8	1	Contrast imaging 1

*ERCP* endoscopic retrograde cholangiopancreatography

*EPS* endoscopic pancreatic stenting

#### Pancreatitis after congenital duodenal atresia/stenosis surgery

In four patients with pancreatitis after congenital duodenal atresia/stenosis surgery, it was difficult to identify the main papilla during the ERCP.

**Fig. 3:**
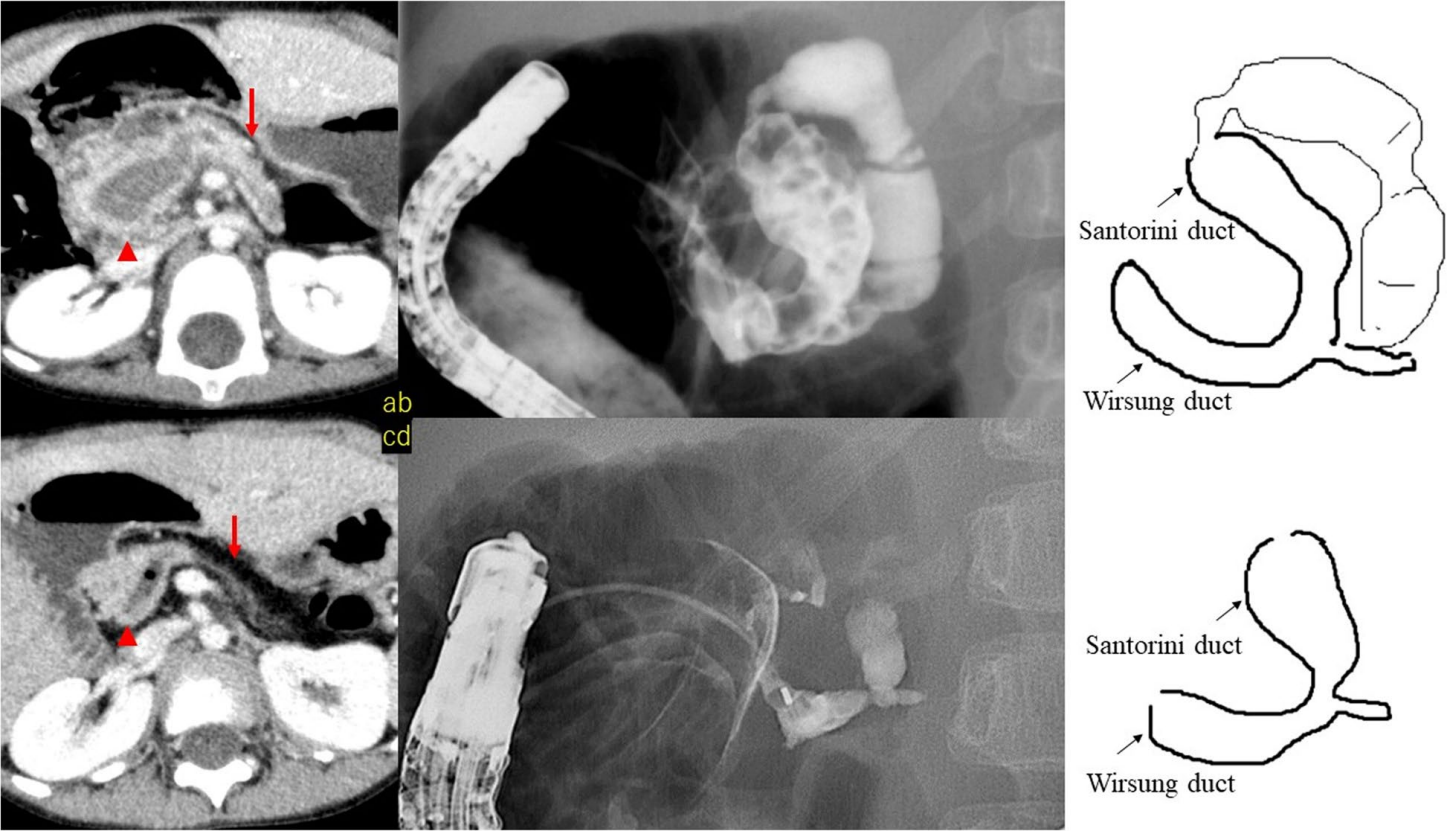
Case 1. Computed tomography (CT) and endoscopic retrograde cholangiopancreatography (ERCP) images of a 3.1-year-old patient are shown in (**a**) and (**b**). Pancreatic stones were found in the pancreatic head, and the pancreatic duct was dilated. CT and ERCP images at 6.3 years of age are shown in (**c**) and (**d**). Atrophy of the tail of the pancreas and decreased exocrine function were also observed. Several ERCP attempts were in vain; thereafter, Longitudinal pancreaticojejunostomy with coring-out of the pancreatic head (Frey’s procedure) was performed at 7-years of age, and there has been no occurrence of pancreatitis since. Arrowhead: pancreatic head, pancreatic duct; Arrow: pancreatic tail

Case 1 was a 3.1-year-old male patient in whom ERCP revealed pancreatic stones and dilatation of the pancreatic duct in the pancreatic head and for whom stone removal was performed. The pancreatic endocrine and exocrine functions of the patient were followed-up on an outpatient basis and, at 6.3 years of age, the patient was found to have decreased exocrine function and atrophy of the pancreatic body and tail. The atrophy was considered to be due to impaired pancreatic fluid drainage. Since papillary balloon dilatation had no effect, placement of a pancreatic duct stent in the pancreatic duct of the pancreatic head was considered to have a high probability of causing long-term withdrawal. The patient underwent pancreatic head and pancreaticojejunostomy at the age of 7 to preserve residual pancreatic function. Thereafter, no postoperative pancreatitis was observed (Fig. 3).

**Fig. 4:**
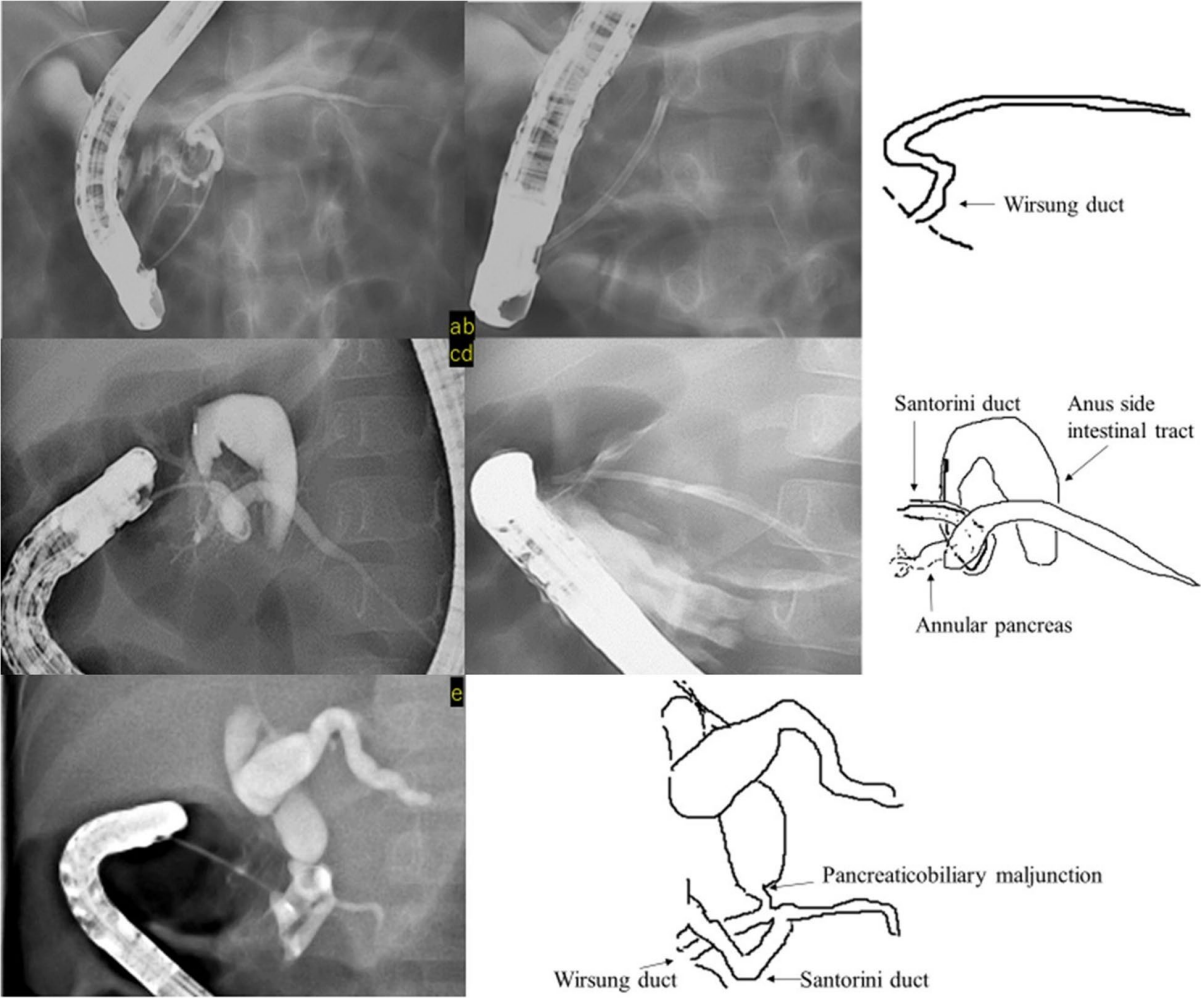
**a**, **b**: Case 2 - Pancreatic duct stent insertion into the caudal pancreatic duct from the main papilla for flexion of the Wirsung duct (**c**, **d**): Case 3 - Contrast-enhanced imaging of the accessory papilla: insertion of a pancreatic duct stent into the caudal pancreatic duct (**e**): Case 4 - Contrast-enhanced imaging of the accessory papilla: angulation of the Santorini duct, and pancreaticobiliary maljunction and choledochal duct dilatation were observed

Case 2 showed flexion of the Wirsung duct, and a pancreatic duct stent was placed from the main papilla to the caudal pancreatic duct. Pancreatic stone removal was only performed during the first surgery. Removal of the stent resulted in recurrence of symptoms; thus, the pancreatic duct stent was replaced regularly and the patient was followed-up. Recurrence of symptoms has not been observed for 13 months after stent placement (Fig. 4a and b).

In Case 3, the endoscope could not be inserted into the main papilla and pancreatic stones were not found in the initial ERCP. In addition, we confirmed that drainage of contrast media from the main papilla and accessory papilla was good. However, due to repeated pancreatitis, a dominant dorsal duct was suspected, and a pancreatic duct stent was placed in the caudal pancreatic duct from the accessory papilla. Although regular stent replacement was required thereafter, the patient has been free from pancreatitis for 19 months after the initial stent placement (Fig. 4c and d).

In Case 4, the main papilla could not be identified in the ERCP at the age of 1 year and 8 months, and contrast imaging was performed from the accessory papilla. Pancreaticobiliary maljunction, dilatation of the common bile duct and angulation of the Santorini duct were observed. The patient underwent laparoscopic extrahepatic bile duct resection at the age of 1 year and 10 months, and no recurrence of symptoms has been observed for 4 years (Fig. 4e).

In two patients who underwent accessory papilla imaging, the accessory papilla was regarded as the main papilla during ERCP, and contrast imaging was performed. Three of the four patients had an annular pancreas (Figs. 3 and 4), and it was difficult to visualize the path of the pancreatic duct, even with contrast imaging. In Cases 1 and 3, the path of the pancreatic duct could only be detected on repeated ERCPs.

#### Chronic pancreatitis

**Fig. 5:**
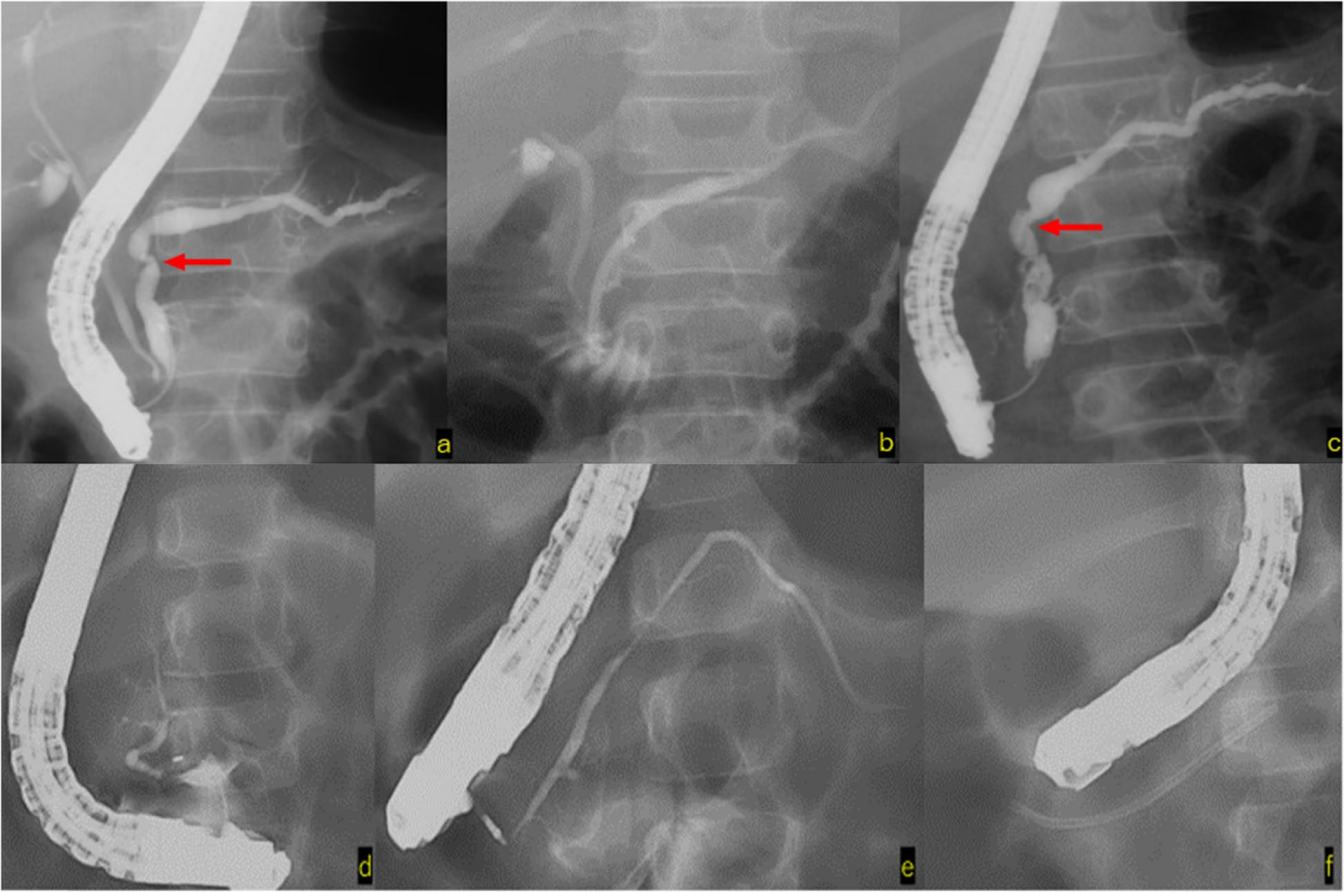
Case 5 (**a**, **b)**: At the time of the initial endoscopic retrograde cholangiopancreatography, stenosis was found in the main pancreatic duct, as seen in the figure. Pancreatic duct stent placement was performed. **c** Improvement of stenosis observed at the time of pancreatic duct stent removal. No recurrence of pancreatitis was observed for 14 years and 5 months after stent removal. Case 9 (**d**) Contrast imaging of the main papilla shows disruption of the Wirsung duct (**e**) Contrast imaging of the caudal pancreatic duct through contrast imaging of the accessory papilla (**f**) Stent placement from the accessory papilla to the caudal pancreatic duct Endoscopic minor papilla sphincterotomy was performed, and no recurrence of pancreatitis was observed for 15 months

All three patients with chronic pancreatitis had diffuse dilatation and wide irregularities involving the pancreatic duct, and pancreatic duct stents were emplaced. EPS was performed repeatedly; however, all patients eventually underwent pancreatic stent removal and were followed up without recurrence of pancreatitis (Fig. 5).

In Case 5, ERCP was performed 6 times, and the stenosis of the pancreatic duct was alleviated by placing a stent. No recurrence of pancreatitis has been observed for 14 years and 5 months after stent removal (Fig. 5a, b and c).

#### Pancreatic divisum and Others

Of two patients with pancreas divisum, it was difficult to insert a stent into the accessory papilla in Case 8 and only contrast imaging was performed; the patient is currently being followed-up on an outpatient basis. In Case 9, a pancreatic duct stent was placed from the accessory papilla to the caudal pancreatic duct, and the patient was confirmed to have no symptoms of pancreatitis, after which endocscopic minor papilla shincterotomy was performed. Fifteen months have passed since the endocscopic minor papilla shincterotomy was performed; however, no recurrence of symptoms has been observed (Fig. 5d, e and f).

In one patient with autoimmune pancreatitis, two ERCP procedures were performed. At the time of initial diagnosis, stenosis of the bile duct and pancreatic duct was found and a biliary stent was placed. Subsequently, steroidal therapy improved the stenosis, the stent was removed, and confirmatory contrast imaging was performed.

Three patients with hereditary pancreatitis and recurrent pancreatitis were found to have no anatomical abnormalities of the pancreatic duct on contrast imaging and were followed up in the pediatrics department.

## Discussion

ERCP appears to be technically difficult and challenging in children because of their narrow airways and smaller anatomical dimensions compared to adults [5]. Improved designs of endoscopes and associated accessories have facilitated the development of ERCP procedures in children [1]. We found that diagnostic and therapeutic ERCP procedure could be successfully performed in childhood, even at three months of age. The only severe complication noted after ERCP was common bile duct injury; there were no cases of severe pancreatitis, bleeding, or perforation.

Our complication rate 1.5% (1/65) was lower than 7.7% incidence typically reported in pediatric large case series [6, 7]. Pediatric ERCP was performed by the most skilled gastroenterologist in our institution, and the use of PJF-7.5 (tip outer diameter, 7.8 mm; channel diameter, 2.0 mm; Olympus) may have avoided unnecessary pressure on the pancreatic duct and thus avoided post-ERCP pancreatitis.

Endoscopic biliary drainage with a temporary stent is adequate for symptomatic relief of CBD. We previously reported that endoscopic short-tube stenting is beneficial in children because it does not require tube management [3]. In our present study, ERCP for biliary tract disease was highly successful and effective. Four patients had unsuccessful stenting, but one patient had symptomatic improvement with guidewie manipulation. Thus, three (5.8%) of the 52 biliary disease cases failed to be treated by ERCP owing to difficulties encountered in cannulation, when the narrow segment was right-angled, or the body size of the patient was too small for the procedure. Percutaneous transhepatic biliary drainage may be useful when stent tube placement is unsuccessful. It has been reported that early biliary decompression should be performed when symptoms persit, but no definite protocol has been established [3, 8]. It is not necessary to perform ERCP in all symptomatic CBD patients, as symptoms may improve with fasting alone. Although there is certainly no statistical evidence to suggest the usefulness of our current treatment strategy for preoperative management of CBD (Fig. 1), our strategy is very safe and effective in carefully selecting patients for ERCP with few complications and safe surgery.

In this study, patients who underwent ERCP for determination of the cause of and treatment for pediatric pancreatitis were also evaluated. ERCP was useful for understanding pancreatic duct anatomy. MRCP can reportedly be evaluated in the same way as ERCP [9]; however, in our experience, the images were unclear and difficult to evaluate. ERCP evaluation in children should be strongly considered because minor artifacts, as seen on MRCP, make evaluation difficult.

We reported four cases of pancreatitis after congenital duodenal atresia/stenosis surgery. Duodenal atresia and stenosis are often accompanied by pancreaticobiliary anomalies due to developmental reasons [10–12]. Growth may also lead to impaired pancreatic drainage due to ductal angulation. Detailed examination of pancreatitis after duodenal atresia associated with an annular pancreas can lead to the diagnoses of pancreaticobiliary maljunction, pancreas divisum, and CBD requiring surgical treatment in some patients [10–12]. Since a wide variety of anatomical abnormalities have been observed, there is a need to carefully search for underlying causes and to develop treatment strategies for pancreatitis after congenital duodenal atresia/stenosis surgery. In Case 2, a pancreatic duct stent was emplaced because the Wirsung duct was bent, and in Case 3, a pancreatic duct stent was placed in the dominant dorsal duct from the accessory papilla. These results suggested that stenting of the pancreatic duct should be useful for improving the angulation and drainage of the pancreatic duct.

In our study, EPS for chronic pancreatitis improved the stenosis that had caused acute exacerbations and, eventually, patients were followed up after repeated stenting without recurrence of pancreatitis. Endoscopic treatments for chronic pancreatitis have been reported to be effective, and favorable results have recently been reported with EPS [5, 13]. EPS can be performed safely even in pediatric patients, and is often the first choice for endoscopic treatment.

In addition, the indications for endoscopic minor papilla sphincterotomy can be decided by placing a pancreatic duct stent in patients with pancreatic divisum and setting the follow-up period. Endoscopic minor papilla sphincterotomy encompasses risk for certain complications such as perforation, bleeding, and acute pancreatitis, and incomplete incision may result in scar stenosis and impaired drainage [14]. Since there is no evidence recommending the direction and extent of the incision, the policy adopted at our institution is to consider minor papillotomy in patients aged 2 years or older (the JF-240 body size that allows the endoscope to be inserted). Thus, the indications for papillotomy should be carefully considered. These experiences suggest that our diagnosis and treatment strategies for pancreatitis in children (Fig. 2) are effective.

In conclusion, ERCP and transendoscopic therapy are sufficiently feasible in pediatric patients and should be actively introduced for the investigation and treatment of pancreaticobiliary diseases.

## Data Availability

The datasets generated and/or analyzed during the current study are available from the corresponding author on reasonable request.
